# Moral injury in healthcare workers: causes & interventions

**DOI:** 10.1093/bmb/ldag011

**Published:** 2026-03-14

**Authors:** Bethany Croak, Danielle Lamb, Rupa Bhundia, Anne-Marie Rafferty, Neil Greenberg, Sharon A M Stevelink

**Affiliations:** Department of Psychological Medicine, King’s College London, Weston Education Centre, Cutcombe Road, London, SE5 9RJ, United Kingdom; Department of Applied Health Research, NIHR ARC North Thames, University College London, Upper 3rd Floor, Royal Free Hospital, Rowland Hill Street, London, NW3 2PF, United Kingdom; Department of Psychological Medicine, King’s College London, Weston Education Centre, Cutcombe Road, London, SE5 9RJ, United Kingdom; Department of Psychological Medicine, King’s College London, Weston Education Centre, Cutcombe Road, London, SE5 9RJ, United Kingdom; Department of Psychological Medicine, King’s College London, Weston Education Centre, Cutcombe Road, London, SE5 9RJ, United Kingdom; Department of Psychological Medicine, King’s College London, Weston Education Centre, Cutcombe Road, London, SE5 9RJ, United Kingdom

**Keywords:** moral injury, healthcare workers, COVID-19, mental health

## Abstract

**Background:**

Moral injury (MI), characterized by psychological distress from morally transgressive events, has been predominantly studied in military personnel but has gained increased attention in healthcare workers (HCWs) since the COVID-19 pandemic.

**Sources of data:**

In this review, we narratively synthesize literature on the causes, risks, consequences, and interventions of MI in HCWs.

**Areas of agreement:**

There is consensus that the COVID-19 pandemic presented HCWs with unique challenges such as fear of infection and patients dying without family, which increased the risk of MI in HCWs.

**Areas of controversy:**

Broader healthcare experiences, not unique to a pandemic, are less well understood. In this review, we discuss evidence of such experiences including restrictive practices in psychiatric settings and experiences of discrimination.

**Growing points:**

Recent studies have highlighted the importance of addressing MI through organizational change, training, and peer support initiatives. Emerging evidence also underscores the need to consider broader systemic factors, such as workplace culture and leadership, in mitigating MI.

**Areas timely for developing research:**

Future research should focus on longitudinal studies to explore in more detail risk factors for MI in HCWs. Additionally, there is a need for robust evaluations of interventions to prevent and treat MI and related disorders, including randomized controlled trials. Investigating the morally injurious effects of systemic issues like understaffing is particularly urgent as the field evolves beyond pandemic-specific challenges.

## Introduction

Depictions of human beings battling morally challenging decisions have long been documented in Greek mythology, novels, and accounts of war. The term moral injury (MI) was first coined by a military psychiatrist, Jonathon Shay [1], following the Vietnam War to capture the distress experienced by soldiers placed in highly morally challenging situations. MI and, more broadly, moral distress, have been defined in many different ways, with a recent proposal to consolidate definitions as follows: ‘Moral distress: Distress that arises from a personal experience that disrupts or threatens: (a) one’s sense of the goodness of oneself, of others, of institutions, or of what are understood to be higher powers, or (b) one’s beliefs or intuitions about right and wrong, or good and evil’. [2].

Morally challenging situations can occur when someone perpetrates (commission), fails to prevent (omission), or bears witness to actions that transgress their moral codes, beliefs, and values [3]. Additionally, the causes of MI also include events involving betrayal, often by a trusted other [4]. Together, such events are referred to as potentially morally injurious events (PMIEs) and may involve accidental deaths in war or military leaders failing to support a distressed team member appropriately. Throughout this review, the authors have sought to mirror the terminology used in the studies discussed, with ‘moral injury’ typically referring to quantitative studies that operationalize the construct using scales measuring exposure to PMIEs, such as the Moral Injury Events Scale [5] and moral distress used when experiences are described qualitatively.

Traditionally confined to military personnel, there has been growing recognition in the last few years that potential MI is an occupational hazard for different groups of workers, including healthcare workers (HCWs), particularly in the wake of the COVID-19 pandemic [6]. The majority of studies into MI among HCWs are recent, mostly emanating from the COVID-19 pandemic era (March 2020–present). In the UK, 28% of HCWs reported experiencing PMIEs at work during the first year of the pandemic (2020–21) [6]. Reports of MI in HCWs have originated from many countries, including the UK, USA (US), Iran [7], China [8], and South Africa [9]. An international meta-analysis showed the pooled prevalence of MI in HCWs from incidents arising from their work, measured during the COVID-19 pandemic, was 45% [10], although, notably, there is no accepted threshold above which someone can be said to be experiencing an MI, as it is not a defined psychiatric disorder.

Most of our understanding of the types of PMIEs HCWs face or the systemic issues that cause them is through the lens of the pandemic. However, there is also some evidence relevant to MI in HCWs outside of a pandemic context, which this review will also discuss. MI theory and measurement have largely been developed in military and veteran contexts, and the application of these models to healthcare work remains emergent. As a result, the extent to which existing conceptual models fully capture the aetiology, mechanisms, and outcomes of MI in diverse healthcare roles and contexts is uncertain. We therefore interpret the emerging literature cautiously and highlight priorities for healthcare-specific theory development and hypothesis-driven research.

## Materials and methods

This review adopted a narrative synthesis approach to summarize and interpret the existing literature on MI among HCWs. Relevant peer-reviewed studies were identified through targeted searches of major academic databases (PubMed, Medline, PsycINFO, and Web of Science) and through citation tracking of key papers in the field. Studies were included if they examined MI or potentially morally injurious experiences among HCWs in any clinical setting. The literature was reviewed iteratively, with papers selected on the basis of relevance to the review aims rather than formal quality appraisal, consistent with narrative review methodology [11].

## Results

### Causes of moral injury in healthcare settings: COVID-19-specific

#### Potentially morally injurious events relating to COVID-19 illness and death

In the early stages of the COVID-19 pandemic, scientists knew little about the virus or its impact on the body. This left HCWs without sufficient knowledge or protocols on how to prevent infection or treat COVID-19. This uncertainty may have led to HCWs feeling they failed to prevent deaths or led to them bearing witness to upsetting events that they felt unable to control; experiences have been described as PMIEs by HCWs [12].

Another unique element of the COVID-19 pandemic was the restrictions on visitors. For the first time in many HCWs’ careers, they were caring for patients who were cared for, and, in some cases, died, without their friends and families around them. The responsibility of being the only person allowed at a patient’s deathbed, informing the patient’s family over the phone, and being the family’s only means of contact with the patient via video call was reported to be morally distressing for HCWs in a qualitative study [12].

Other pandemic-specific PMIEs have been investigated in relation to MI. For instance, HCWs who were worried about contracting COVID-19 [13], were redeployed [6], or had a colleague die of COVID-19 [6] were at greater risk of MI, as measured by The Moral Injury Events Scale [5].

#### Potentially morally injurious events relating to community behaviour

Outside of the COVID-19 wards, the public’s reaction to the pandemic impacted HCWs. HCWs became the focus of national campaigns; for example, in the UK, slogans such as ‘'stay at home, save lives and save the NHS’ were used to encourage compliance with lockdowns, and clapping for HCWs at a set time each evening was encouraged to show support. This perpetuated a narrative that HCWs were ‘heroes’ and imagery to that effect was used in murals and advertising. This narrative was not isolated to the UK. In a qualitative study, HCWs in the USA reported struggling with this ‘hero’ image whilst feeling they had let down their patients, leading them to feel shameful and guilty [12]. Other studies have found HCWs had mixed feelings about the clapping campaign and the hero label, stating they felt it distracted from the severity of the pandemic and the scarce resources available to them to perform their job to the highest possible standard [14].

### Organizational and systemic factors

The overwhelming number of patients requiring a limited supply of equipment (e.g. ventilators) and the need for enhanced personal protection equipment (PPE) for HCWs to protect them from the virus caused many hospitals to run out of resources quickly. This left HCWs vulnerable to making difficult decisions on how to share resources. Indeed, two studies found that HCWs who reported inadequate or a lack of PPE [6, 15] had increased odds of MI.

Throughout the COVID-19 pandemic, frequent changes in lockdown regulations and treatment guidelines frustrated many HCWs, who felt these lacked scientific backing. Some felt they had let patients down, whilst others perceived betrayal by senior management. For instance, some HCWs believed government messaging undermined the severity of COVID-19, which they were witnessing at work, and others felt lockdowns were disproportionate and were likely to have long-lasting impacts on mental wellbeing [16]; these contrasting views likely threatened team cohesion. Confidence in the UK government was undoubtedly undermined by high-profile government figures breaking the rules themselves [17], with many HCWs feeling betrayed by events such as ‘Partygate’, where the UK Prime Minister was caught having parties during lockdown. Those HCWs who felt unsupported by, or who lacked trust in, the government reported higher levels of moral distress [16], highlighting the impact of political context on PMIEs in healthcare.

The development of COVID-19 vaccines eased the burden on healthcare services, offering some protection against COVID-19 and preventing many from serious illness. For some HCWs, religious or ethical objections underpinned their decision to receive the vaccine, especially among HCWs from an ethnic minority background [18]. Vaccine hesitancy also stemmed from mistrust in government and pharmaceutical companies. This was especially salient for HCWs from Black ethnic groups who felt their communities had been abused or mistreated in vaccine trials in the past [18]. In the UK, mandatory vaccination for HCWs (later reversed) sparked controversy [19]. Although no study to our knowledge has measured the relationship between this policy and MI, it may have been experienced as ethically challenging and may have contributed to mistrust among some HCWs, particularly those from racial and ethnic minority backgrounds who, in the UK, make up 26.5% of the healthcare workforce [20].

### Causes of moral injury in healthcare settings: non-COVID-19 experiences

COVID-19 experiences are not the only PMIEs faced by HCWs, although it is important to note much of the MI-related research commented on in this section was carried out during the pandemic.

Whilst all HCWs may experience PMIEs, certain specialities are particularly likely to be exposed to them. For instance, staff in inpatient mental health settings may be required to use mechanical and chemical restraints, and they can be victims of patient aggression or violence. In a study of HCWs working in a secure mental health unit, violence and restrictive practices were associated with MI, although these associations were only significant in women from White ethnic groups [21]. Similarly, a qualitative study reported that nurses in psychiatric settings described the use of mechanical restraint as morally distressing and associated with experiences of MI [22].

Staff in physical health settings are also likely to experience PMIEs. For example, all participants (*n* = 14) in a qualitative study of Australian midwives described experiencing workplace events that compromised their moral values, including working with colleagues whose practices or values they believed had caused patients distress and staffing resources preventing mothers-to-be from having midwife-led care. Some midwife participants also discussed how pregnancy termination potentially contravened their personal beliefs [23].

In addition to PMIEs arising from medical dilemmas or the provision of care, cultural factors can also place HCWs at risk of MI. For instance, HCWs from Black, Latino, and mixed ethnic groups in the USA who witnessed prejudice and bias by colleagues when treating patients of colour and faced discrimination themselves were at increased risk of MI [24]. It thus appears HCWs from ethnic minority groups are at increased risk of experiencing MI not just from the clinical work they do but as a result of racial and ethnic inequality and prejudice they experience at work. These experiences represent PMIEs in which distress arises from being subjected to, or witnessing, morally harmful actions by others within systems of power rather than from one’s own perceived transgression. Related exposures in healthcare may include institutional betrayal (e.g. perceived lack of protection, support, or fairness), coercive organizational practices, or moral conflicts arising from enforcing policies experienced as harmful. Although these themes are increasingly reported, they remain under-represented in the literature.

There are likely more examples of PMIEs outside of a pandemic context, but the literature is currently skewed towards the pandemic. Therefore, research should not stop exploring MI in HCWs after the pandemic but continue to understand how routine practices in healthcare may be morally injurious to HCWs.

### Organizational factors

In this section, we discuss systemic and organizational issues beyond the COVID-19 pandemic, although arguably these issues may have been exacerbated by the pandemic.

Whilst organizational culture, environment, and values are pertinent to all workers’ wellbeing, these factors are of heightened importance in psychologically charged and demanding roles such as those in healthcare. An effective healthcare organization has been described as one that promotes speaking up about concerns without fear of retaliation (important when witnessing PMIEs), has forums with leaders to share concerns, has crisis response policies, and offers psychological support for staff [25]. Unsurprisingly, research has found increased organizational effectiveness is associated with a lower risk of MI [25]. Additionally, research has found an organizational culture in which individuals had little control over job demands/duties, where employees were not rewarded, and targets and outcomes were prioritized over ethical values increased the risk of MI in HCWs [26].

In such environments, HCWs may feel unable to raise ethical concerns, disclose distress, or challenge practices they experience as morally troubling, particularly within hierarchical organizational structures where power and job security are unevenly distributed. Fear of professional, legal, or reputational consequences may further discourage disclosure, contributing to isolation, concealment, and distrust in organizational and support systems. Together, these findings highlight that the risk of MI is shaped not only by exposure to PMIEs but also by the extent to which healthcare organizations foster psychological safety, ethical dialogue, and supportive leadership.

In a qualitative exploration of HCWs’ experiences during the COVID-19 pandemic, HCWs described feeling ill-equipped to deal with the crisis and/or under-supported. Participants attributed this partially to prepandemic systemic issues such as government underfunding, understaffing, and prepandemic strained relationships between employees and managers. The pandemic then exacerbated these issues, meaning participants felt unable to provide adequate care and felt forced to act beyond their competency level [16].

HCWs who felt unsupported and thus betrayed by their managers and colleagues had poorer mental health outcomes [27]. Another study found that a less supportive work environment at baseline predicted higher scores on the Moral Injury Event Scale at 3 months [28]. Similarly, those who reported less leadership support were more likely to experience MI [13].

### Staff groups at higher risk

The majority of literature that investigates MI risk factors is cross-sectional, although the few longitudinal studies provide a better understanding of the predictors of MI following a PMIE [13, 28, 29]. However, to the best of our knowledge, there are no studies that have measured baseline characteristics before the pandemic and predicted the new onset of MI during the COVID-19 pandemic or beyond.

### Socio-demographic characteristics

There is some evidence to suggest certain socio-demographic characteristics put individuals at greater risk of MI. For example, being younger [30–32], being a woman [33], and HCWs from an ethnic minority group were more likely to report having behaved in a way that violated their moral code (i.e. acts of commission or omission) [30].

### Personality

Some studies have explored personality traits or type and MI risk. In a study of HCWs working in China, scores on three subscales of The Light Triad Scale [34] were negatively correlated with MI [35]; the three dimensions of a light personality style are Kantianism (treating people as ends in themselves), Humanism (valuing the dignity and worth of each individual), and Faith in Humanity (believing in the fundamental goodness of humans) [34]. Another personality trait found to be protective for MI was higher levels of self-esteem [36]. Conversely, a study of US HCWs found no difference in MI scores between those who exhibited a high stress reaction and low wellbeing on a personality measure compared to those with low stress reaction and high wellbeing [37].

### Psychological resilience and pre-existing mental health difficulties

Psychological resilience, often discussed in mental health and MI literature, refers to maintaining or returning to healthy functioning after adversity [38]. Whilst some cross-sectional studies suggest resilience reduces MI risk [33, 39, 40], longitudinal studies are needed for confirmation. Two longitudinal studies found no effect of resilience on the risk of MI (they measured four time points over a period of 6 months and three timepoints over 3 months, respectively) [13, 28].

On the other end of the spectrum, a longitudinal study of HCWs found that a prior diagnosis of depression and experiences of stress were associated with higher levels of MI over a 6-month period [41]. Similarly, prior mental health adversity was associated with MI related to witnessing a PMIE 6 months later [13].

### Occupational characteristics

Studies comparing MI prevalence across occupational roles suggest nurses are at higher risk than doctors or support staff, such as technicians [13, 32, 42, 43]; interestingly, one study found being a nurse predicted MI related to witnessing a PMIE, but it was not associated with MI caused by acting against one’s morals or failing to act [13]. However, a high proportion of nurses are women [44], and whilst these studies include gender in a multivariate analysis, a more in-depth analysis would be beneficial to understand the interplay between gender, occupational role, and MI. A US study found clinical staff more likely than nonclinical staff to report betrayal-related PMIEs [45]. Similar to older age being protective, a greater number of years of experience in healthcare was associated with a reduced likelihood of MI [33]. However, it is unclear if this is a ‘healthy worker effect’, where those who are negatively impacted by PMIEs have left the healthcare workforce.

### Possible consequences of moral injury

MI is not a formal diagnosis, but individuals who experience MI can experience a range of negative reactions to PMIES, including shame, anger, disgust, and guilt. MI may also lead to changes in the way people view themselves or the world, including developing maladaptive coping methods such as substance misuse [46]. Across multiple occupations before the pandemic, experiencing PMIEs has been associated with an increased risk of post-traumatic stress (PTSD), depression, and suicidality [4].

In the first wave of the pandemic, MI was found to increase the risk of common mental disorders (CMDs), PTSD, and alcohol misuse in UK HCWs [47]. An international meta-analysis found MI was associated with an increased likelihood of depression, anxiety, PTSD, burnout, and suicidal ideation [48]. More specifically, omission of PMIEs, including feeling unable to provide adequate care, was associated with increased suicidal ideation in HCWs [49]. Although, as described previously, this relationship can be bi-directional [13, 29]. Nevertheless, in 2024, mental disorders were the most common reason for sickness absence in the UK’s healthcare service [50]; therefore, mental distress from MI is a concern for staffing levels and, subsequently, quality of patient care.

Qualitative studies suggest that MI among HCWs is associated with various occupational outcomes, though quantitative evidence remains limited. In one such qualitative study, participants who had experienced PMIEs described a loss of identity as a nurse [51], whilst another found HCWs in Spain experienced reduced confidence in their ability after focusing solely on respiratory patients [52]. Lastly, evidence suggests experience of PMIEs increases the likelihood of HCWs leaving healthcare [53–55]. Staffing levels can have subsequent impacts on waiting lists and patient care, which not only means HCWs cannot provide the care needed, which could be morally injurious, but it has also been associated with an increased risk of patient mortality [56]. Given the wide implications of occupational outcomes, further research, specifically quantitative, is needed to establish if MI is associated with other occupational outcomes, such as sick leave.

## Discussion

### Can we prevent moral injury?

Although postexposure mitigation strategies for PMIEs are important, preventative strategies that aim to reduce exposure to, or the impact of, PMIEs among HCWs are optimal. Many of the individual risk factors identified, e.g. sex, are not modifiable. Therefore, preventative strategies should not put the onus on the individual but on the organization. The research identified in this review [16, 25] highlights systemic issues within healthcare services, which, although exacerbated by COVID-19, existed pre-pandemic and continue to do so. These include understaffing and underfunding. In the UK in 2023, 8.4% of NHS posts were unfilled [57] and in March 2024, 6.3 million people were on a waiting list for treatment [58]. Addressing some of these systemic issues, which staff might view as betrayal, may be associated with improved working conditions for HCWs and greater capacity to provide high-quality care and may, in turn, reduce the likelihood of MI. A preventative approach may be more sustainable than current temporary fixes, such as the use of bank and agency staff.

However, we recognize that stressors, including PMIEs, are also likely to arise within an ever-changing work environment; people now think and feel differently, hold different values, and are required to make decisions that can impact the lives of others in ways that differ from 20 or more years ago. Such changes should not necessarily be ‘over medicalized’, but instead, we should focus on creating working environments that can better prepare HCWs to encounter them.

One potential approach is to prepare staff for realistic outcomes, which may help them to process difficult events when they happen. For example, whilst resuscitation training often leads to the CPR mannequin surviving, many cardiac arrests, especially outside hospitals, result in death. It follows that all resuscitation students should be taught that the likelihood of resuscitation ending in death is high despite their best efforts. Fostering thoughts like ‘the person is dead, but I might revive them’ may be more psychologically protective than ‘if I follow the resuscitation procedure properly, then they won’t die’. Such approaches need further research, but existing MI literature suggests overly optimistic expectations may increase the risk of MI [59].

Creating opportunities for staff to reflect and discuss PMIEs through supervision, peer support, or similar sessions may help attenuate the impact of a PMIE. Reflective practice sessions show promise in reducing MI in HCWs, and there are calls to integrate peer support groups into medical training [60–62]. Informal peer support that happens naturally within teams is important to wellbeing in general; whilst some found cohesion through a shared experience during the pandemic, this appeared to deteriorate for some HCWs due to re-deployment and isolation from colleagues. In addition, equipping managers with the skills and confidence to discuss these incidents with their employees and support them may mitigate some of the negative impact of these events. However, much of the evaluation of these practices has been on a small scale [60, 61]; larger, randomized controlled trials are needed. Similarly, many of these interventions have focused on clinical staff; interventions should include nonclinical staff, such as healthcare assistants, who also may face PMIEs. Taken together, these approaches emphasize the importance of moral repair at a collective and organizational level, through leadership, peer support, and workplace cultures that foster trust, belonging, and shared values, rather than relying solely on individual psychotherapy.

### How to treat moral injury and moral injury–related mental disorders

Unfortunately, there will be instances where PMIEs lead to persistent MI. Whilst MI is not a formal diagnosis, given its association with the development of mental disorders [47, 48] and the potential link with leaving one’s job [53–55], it makes sense to find ways to help resolve MI. Although distinct from PTSD, some have theorized that trauma-focused PTSD treatments such as Prolonged Exposure (PE) and Cognitive Processing Therapy (CPT) might be effective in treating MI [63]. In CPT, patients are encouraged to address beliefs such as ‘I am unforgivable’, which underlie the guilt and shame experienced by those with MI. However, whilst trauma-focused treatments such as CPT and EMDR explicitly address maladaptive appraisals related to guilt and shame, guilt—particularly when rooted in moral concerns—can nonetheless remain clinically challenging and may not be fully resolved through exposure-based mechanisms alone [64].

In response to these challenges, some have proposed that existing trauma-focused treatments can be adapted to address MI-related difficulties as showcased by a case study in which cognitive behavioural therapy (CBT) was adapted to treat MI-related PTSD in an HCW [65]. In parallel, other researchers have developed interventions specifically designed to address MI, which have shown promise in military populations [66]. However, the evidence base for both approaches remains limited, and further research is needed to establish their effectiveness and applicability in HCWs.

In addition to the treatment model, the individuals who deliver it might also need to be different. MI is rooted in morality and ethics, which makes it somewhat unique with regard to the standardized treatment of psychological disorders. Therefore, treatment by mental health professionals who are not well acquainted with the topic may not be appropriate. In addition, individuals may be reluctant to disclose acts of commission, in particular due to concerns about legal consequences, and clinicians can be unsure about the limits of confidentiality in these cases [67]. It may be that professionals from other disciplines, such as religious leaders like hospital Chaplains, who are adept at such discussions, might be well placed to help HCWs following PMIEs [68].

Due to the nature of healthcare work, there may be some PMIEs that, unfortunately, cannot be avoided, for example, when the law contradicts one’s personal beliefs, such as rules on euthanasia and pregnancy terminations. However, allowing HCWs to process these events in a safe space, such as through peer groups, reflective practice, or debriefs, may mitigate the likelihood of MI [16, 69]. Trauma risk management (TRiM) is a formalized peer support process common in the UK’s National Health Service (NHS). It aims to actively monitor trauma-exposed HCWs, facilitate workplace support, and encourage early referral of HCWs to professional support if their mental health does not improve [70]. However, TRiM and similar models rely heavily on adequate training, organizational buy-in, and the availability of onward support, which may be inconsistent across settings and over time. Furthermore, funding for mental health support initiatives introduced during COVID-19, such as the Wellbeing Hubs, has been substantially reduced only a few years after the pandemic. This risks undermining continuity of care and limits the ability to evaluate longer-term effectiveness. Taken together, these issues highlight a pressing need for sustained funding, longitudinal research, and robust evidence-based practice to support HCWs exposed to PMIEs.

In addition, opening dialogue between employees and managers might help repair the relationships that have broken down and allow healing to take place in the case of feelings of betrayal [71]. These conversations can also prevent similar situations from recurring and repair trust, which can be helpful in recovering from MI [71]. This can be done at an organizational level and a government level, for example, with the UK COVID-19 pandemic public inquiry.

In summary, although several approaches have been suggested to address MI and associated mental health difficulties, the current evidence base remains underdeveloped. Intervention studies have largely focused on military populations, and it remains unclear to what extent these findings generalize to HCWs or other civilian occupational groups. Further high-quality research is needed to strengthen the evidence base and inform evidence-based practice in non-military contexts.

### Gaps in the literature and future directions

A key limitation of the current evidence base is the relative lack of healthcare-specific theoretical grounding for MI. Much of the conceptual framing has been derived from veteran-based models, yet it remains unclear how well these translate to healthcare work, where moral stressors are often cumulative, shaped by organizational constraints, and may involve nonagentic experiences (e.g. witnessing others’ transgressions, systemic betrayal, or discrimination). In addition, the two populations differ substantially. Military personnel are mainly male, whereas healthcare services are staffed by a majority of women [44] and a substantial proportion (26%) of HCWs in the UK are from ethnic minority groups [72]. Given the demographic makeup of the healthcare workforce and evidence that these groups may be at greater risk of MI [33] it is important to explore specific PMIEs these staff groups might face. Future research should also explicitly examine the role of cultural and structural factors, including experiences of discrimination, marginalization, and power imbalance within healthcare organizations, in shaping exposure to PMIEs. Further, the field would benefit from developing and testing models that specify mechanisms and generate predictions tailored to healthcare roles, settings, and structural conditions. This would also support clearer differentiation between exposure to PMIEs and MI-related outcomes and improve measurement and intervention development.

Most of the research was conducted during the COVID-19 pandemic, and although some PMIEs such as fear of infection were more pertinent during the pandemic era, other PMIEs that occurred during the pandemic are likely to have long-lasting effects. Relatedly, systemic issues such as underfunding and staffing pressures existed before the pandemic and still prevail today. These systemic issues will likely be compounded in the future by further pressures such as an ageing population. Therefore, researchers should continue to monitor the impact of these pressures. A further limitation is that the healthcare MI literature has tended to prioritize agentic MI (acts of commission/omission) and broad systemic stressors, with comparatively less attention to nonagentic experiences such as witnessing others’ transgressions, institutional betrayal, coercive practices, or direct victimization (including discrimination). Future research should therefore examine whether the psychological mechanisms and outcomes differ between different types of PMIEs.

A further challenge in interpreting the existing evidence base relates to how MI is measured and described. A recurring issue in the literature is the conflation of exposure to PMIEs with MI as an outcome. Although MI is not a formally recognized mental disorder, it is commonly operationalized through self-report measures assessing exposure to events that conflict with an individual’s moral values and the associated moral distress. In practice, individuals are classified as having experienced MI based on endorsement of one or more morally transgressive events [6]. Consequently, references to ‘increased odds of MI’ often reflect greater exposure to PMIEs or heightened moral distress, rather than the presence of MI as a discrete clinical condition. Whilst this conceptualization is consistent with current methodological practice, it highlights the need for caution in the language used to describe MI and for clarity when interpreting findings.

Finally, we identified very few longitudinal studies in this review and those that were longitudinal in nature rarely had time points longer than 6 months. As a result, the predominance of cross-sectional designs limits causal inference, and observed relationships between individual vulnerabilities, organizational factors, and MI should be interpreted cautiously. Further longitudinal research is needed to clarify temporal ordering, explore potential bidirectional relationships, and examine the longer-term associations between MI and outcomes such as staff mental health, retention, sickness absence, and patient safety.

## Conclusion

In conclusion, MI has historically been viewed as associated with military personnel; however, recent research has highlighted its significant impact on HCWs, especially in the wake of the COVID-19 pandemic. The unique and challenging experiences faced by HCWs during the pandemic, coupled with pre-existing systemic issues such as understaffing and inadequate resources, are likely to have exacerbated the risk of MI in this occupational group. Although initial research has provided valuable insights, there is a need for more longitudinal studies to understand the long-term effects of MI on HCWs, not only on their health and wellbeing but also on patient care and safety and wider organizational performance. Preventative measures, such as realistic preparation, peer support, and reflective practice, also warrant further exploration to mitigate the risk of MI. Furthermore, the development and testing of evidence-based treatments specifically for MI in this population is essential. Addressing these gaps in the literature and treatment provision will be crucial in supporting HCWs and ensuring their wellbeing in the face of ongoing and future challenges.

## Data Availability

No new data were generated for the study, as the review is based on existing published literature, and therefore, data are available within the cited articles and their supplementary materials.
